# Partial protein domains: evolutionary insights and bioinformatics challenges

**DOI:** 10.1186/s13059-015-0663-8

**Published:** 2015-05-19

**Authors:** Lawrence A Kelley, Michael JE Sternberg

**Affiliations:** Structural Bioinformatics Group, Centre for Integrative Systems Biology and Bioinformatics, Department of Life Sciences, Imperial College London, London, SW7 2AZ UK

## Abstract

Protein domains are generally thought to correspond to units of evolution. New research raises questions about how such domains are defined with bioinformatics tools and sheds light on how evolution has enabled partial domains to be viable.

With the rapid expansion in the number of determined protein sequences - over 92 million in UniProt in March 2015 - an ever-increasing number of biologists are using bioinformatics tools for annotation of these sequences. One widely used strategy is to identify occurrences of Pfam families within the sequence of interest [1]. A Pfam family is a multiple sequence alignment of the occurrences of a particular domain both in different species and in different regions of the same protein. The concept underpinning Pfam is that proteins typically comprise one or more domains (regions), each of which is an evolutionary unit that generally has a well-defined biological function. A significant sequence similarity between a query protein and a Pfam family provides the basis for annotations. Two recent articles [2,3] in *Genome Biology* evaluate the implications of having the query sequence only matching part of a Pfam family, which is an intriguing finding, given that a Pfam family is considered to be an evolutionary unit.

## What is a protein domain?

In discussing these articles, it must be emphasized that the definition of a protein domain is complex. One approach to define a protein domain is from a structural perspective. This was highlighted in Wetlaufer’s seminal article [4], where he noted that several protein structures can readily be divided into distinct structural regions (called domains), with few interactions between the domains and few chain crossings between them. The suggestion was that each domain was an independent folding unit. Moreover, certain domains were associated with a particular function, such as NAD binding, and thus these domains were considered an evolutionary unit.

Another approach, currently used in Pfam [1], defines domains from a sequence perspective. This approach aims to identify a section of the protein sequence that shares significant sequence similarity elsewhere in the protein or in other proteins. The use of multiple alignments is central to assessing whether remote sequences are actually similar. In Pfam, the multiple sequence alignment is stored as a hidden Markov model (HMM), which is a statistical formulation that quantifies the archetypal sequence signature that is characteristic of that domain. Underpinning the sequence-based approach is the concept that a domain is an evolutionary unit.

In many proteins, the structural and the sequence-based approaches yield a very similar definition of the domains forming a protein. However, this is not always the case. In Pfam (A Bateman, personal communication), the families are primarily derived based on sequence alignment, but additionally take into account the concept that a Pfam family is likely to be a structural domain. However, as we do not have the structures for all proteins, a Pfam family subsequently could be split once a structure is determined - but of course this takes time. Moreover, the structural definition of a domain involves substantial subjective input, and algorithms are able only to provide guidance. The extent of the problem of defining domains is highlighted in a recent study to map CATH (for ‘class-architecture-topology-homologous superfamily’) [5] to SCOP (‘structural classification of proteins’) [6] domains undertaken in the Genome3D consortium [7]. The principle behind SCOP is that a region has to be seen independently to merit being defined as a domain, and so there is a class in SCOP called multi-domain proteins that has multi-lobal structures. CATH, by contrast, would split the multi-lobal structure into component domains. Only 60% of CATH domains are similar to a SCOP counterpart (the silver standard in Genome3D) (N Nadzirin and C Orengo, personal communication).

## Explanations for a partial match to an archetypal Pfam domain

Two recent *Genome Biology* articles investigated partial matches to Pfam domains. Triant and Pearson [2] show that almost 4% of Pfam domains from a representative subset (RefProtDom2 (RPD2) with 136 families) are shorter than 50% of the length of the HMM of that family. Detailed examination identifies several reasons for these partial matches. The largest fraction of 50% partial domains are split-partials - partials where a complete domain has been split into smaller regions. After inspection, one could reassemble the full-length domain, but a naive user of Pfam might not be aware of the problem. In many other cases, there is strong evidence that this partial alignment is the result of errors in the identification of the protein sequence in the truncated domain, and this is particularly prevalent in eukaryotic sequences from poorly annotated species. Other occurrences of partial domain matches can be explained by the domain being split by an inserted region or an alignment that should extend beyond the matched region, but has failed to do so owing to a weak sequence similarity signal. However, there are a few observed instances (18 out of the 136 Pfam families) where the Pfam HMM corresponds to multiple CATH [5] domains or multiple ‘vector alignment search tool’ VAST+ [8] domains, or both. Thus, the apparent partial match will typically be the result of matching to one of these component domains. This study highlights the challenges in protein annotation and in using sequence families to identify independent structural domains.

A related question is considered by Prakash and Bateman [3]. They identify a few proteins where the protein sequence lacks one-third or more of the residues of the Pfam model. Manual inspection and filtering identifies proteins where the partial match could be explained by gene-prediction error or by other well-recognized effects such as multi-domain proteins. However, after filtering, they identify sequences where there is a partial match to an independently folded domain, and the authors introduce the term 'domain atrophy'. They note that domain atrophy is very rare (0.06% of all Pfam domains), but when it does occur it raises the question of how such a partial domain is able to fold into a functional stable unit. For 75 domains where such atrophy occurs, there is an available structure - either experimental or inferred from the coordinates of a homolog. Detailed inspection shows that often domain-domain or subunit-subunit interactions lead to stabilization of the atrophied domain.

**Figure 1:**
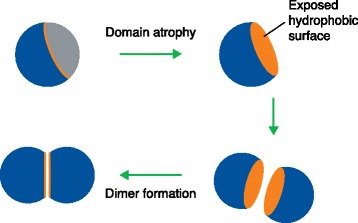
Schematic illustrating how domain atrophy (loss of gray region from blue sphere) can lead to exposure of hydrophobic residues (orange). This exposed hydrophobic surface can subsequently be stabilized by dimerization. See Prakash and Bateman [3] in this issue for detailed examples.

A particularly striking case (illustrated schematically in Figure 1) is the bacterial luciferase domain from *Photobacterium phosphoreum* LuxF [9]. This domain lacks one β-strand and three α-helices from the standard structure of bacterial luciferase that comprises eight buried β-strands forming a β-barrel surrounded by α-helices. One might expect that the fold would be highly unstable. Indeed, the crystal structure of this protein reveals that the monomer has a large hydrophobic cleft that is not buried. However, a homo-dimeric interaction buries this cleft and thus stabilizes the protein. This and the other examples that the authors list highlight the mechanisms by which evolution has managed to ensure that atrophied domains remain viable.

## General implication for protein bioinformatics

The difficulties that can occur in Pfam-based protein annotations can also arise when other strategies are applied, such as in prediction of protein structure. For example, a web-based resource for template-based modelling is available for the community - Phyre2 [10]. These two articles will provide for the users valuable suggestions as to the possible causes for Phyre2 predicting only a partial protein domain. Thus, when interpreting results of bioinformatics resources, biologists need to consider both the possibility of erroneous data and the fascinating diversity of mechanisms that can occur during evolution to deliver biological function.

## Abbreviations

CATH: Class-architecture-topology-homologous superfamily
HMM: Hidden Markov model
NAD: Nicotinamide adenine dinucleotide
RFPD2: RefProtDom2
SCOP: Structural classification of proteins
